# A Retrospective Study of the Relationship Between Hypertension and Vestibular Disorders in Middle-Aged Women With and Without HIV

**DOI:** 10.7759/cureus.34988

**Published:** 2023-02-14

**Authors:** Helen S Cohen, Michael W Plankey, Deanna Ware

**Affiliations:** 1 Otolaryngology - Head and Neck Surgery, Baylor College of Medicine, Houston, USA; 2 Medicine, Georgetown University Medical Center, Washington DC, USA

**Keywords:** dizziness, vertigo, vestibular testing, hypertension, hiv

## Abstract

Background: Patients often conflate the problem of lightheadedness from hypertension (HTN) and vertigo from a vestibular impairment, describing both problems as dizziness. The goal of the study was to learn if there is a relationship between measures of vestibular function and blood pressure.

Methods: This retrospective study consisted of women who participated in a longitudinal study of the human immunodeficiency virus (HIV) and a control cohort of age-matched women without HIV. We used data from the point in time when participants were tested for vestibular functions with bi-thermal caloric tests and cervical vestibular evoked myogenic potentials; the data also included the blood pressure of the participants.

Results: High odds ratios (1.48 to 2.05) suggest a relationship between HTN and vestibular impairment, although the sample size was too small to reach statistical significance.

Conclusion: The data suggest that high blood pressure may be related to vestibular impairments. Clinicians whose patients complain of vertigo and balance disorders consistent with vestibular impairments should consider blood pressure as a related problem during the initial visit.

## Introduction

Vertigo and dizziness have a lifetime prevalence of >20% and are among the most common reasons for patients to visit physicians [1]. Hypertension (HTN) may cause lightheadedness, and impairments of the vestibular system (VNG) may cause vertigo. Patients often describe both lightheadedness and vertigo as dizziness, which can confuse clinicians. The prevalence of HTN is high in people living with HIV (PLWH) and people living without HIV (PLWOH) [2]. Vertigo and lightheadedness have similar prevalences and may co-occur, more so in women than in men [3]. Recent evidence has shown that stage 2 HTN is a predictor of abnormal results on objective tests of the VNG [4]. One study showed that the frequency of abnormal VNG responses did not differ between middle-aged women in both groups - PLWH and PLWOH [5]. In healthy controls and PLWH, people of all ages who did not complain of vertigo had evidence of vestibular impairment, although it was more common in older adults. Because HIV patients are at risk for HTN, and because women are at risk for vestibular impairments, we sought to determine if HTN predicted abnormal VNG results in middle-aged and aging women in both groups - PLWH and PLWOH. One of the authors (MWP) is an investigator with the ongoing Women’s Interagency HIV Study (WIHS) [6] and has access to the relevant database.

## Materials and methods

Subjects

This retrospective study was conducted between February 2018 to March 2019 and included participants from the Georgetown University WIHS site who had previously participated in a study on vestibular functions [5]. The study included WIHS participants who were PLWH as well as age-matched controls (PLWOH subjects) with similar socioeconomic status. They were sent a letter requesting their participation in the previous study and they volunteered to participate. The Institutional Review Board of Human Subjects Research for Baylor College of Medicine and Affiliated Hospitals issued approval H-22229.

Test measures

The VNG included bi-thermal caloric tests with air (calorics) and cervical vestibular evoked myogenic potentials (VEMP). For calorics, eye movements were recorded with infrared video-oculography. The collection of these measures was described previously [5]. The test outcomes were classified as normal or abnormal: bi-thermal caloric weakness (caloric; abnormal, bi-thermal caloric weakness ≥25%), cervical VEMP and bi-thermal caloric test plus VEMP (Caloric + VEMP). 

Blood pressure was measured by a physician assistant using a digital sphygmomanometer. Using published ranges for normal blood pressure [7] HTN was defined as 1) systolic blood pressure >130 mmHg or diastolic >80 mmHg; 2) taking anti-hypertensive medication; 3) diagnosis of HTN by a physician. Other independent measures included HIV status and age in years. 

All testing was performed on outpatients at an academic medical center in a large city. Subjects in the study were seen for blood pressure testing at the Department of Medicine of the WIHS clinic. They were also tested on VNGs by audiologists at the Department of Otolaryngology.

Statistical analyses

This study was a retrospective review of existing data. We generated descriptive statistics and used Fisher’s exact test to test the differences in distribution by HTN and HIV status. We used logistic regression to model the odds of abnormal vestibular outcomes and HTN status, which was adjusted for age and HIV status. All analyses were performed in SAS version 9.4 (SAS Institute Inc., Cary, NC).

## Results

The analyses included 77 female participants (81.8% PLWH/18.2% PLWOH) with a median age of 54.0 years (interquartile range: 49-61). HTN was reported in 59.7% (N=46) of the participants (Table 1).

**Table 1:** Sample Characteristics by HIV Status and HTN Status (N=77) P-values for statistical significance were at least p=0.05. Human immunodeficiency virus (HIV), hypertension (HTN), interquartile range (IQR), sample size (N), people living with HIV (PLWH), people living without HIV (PLWOH), cervical vestibular evoked myogenic potentials (VEMP).

	No HTN N=31			HTN N=46		
	PLWOH N=5	PLWH N=26	p-value	PLWOH N=9	PLWH N=37	p-value
Age (yrs), Median/IQR	59 (55-61)	55 (50-62)	0.98	54 (49-55)	53 (49-60)	0.44
Caloric						
Normal	5 (100%)	22 (84.6%)	1.00	8 (88.9%)	30 (81.1%)	1.00
Abnormal	0 (0%)	4 (15.4%)		1 (11.1%)	7 (18.9%)	
VEMP						
Normal	5 (100%)	26 (100%)	1.00	9 (100%)	36 (97.3%)	1.00
Abnormal	0 (0%)	0 (0%)		0 (0%)	1 (2.7%)	
Caloric + VEMP						
Normal	5 (100%)	22 (84.6%)	1.00	8 (88.9%)	28 (75.7%)	0.66
Abnormal	0 (0%)	4 (15.4%)		1 (11.1%)	9 (24.3%)	

Abnormal caloric, VEMP, and Caloric + VEMP measurements were present in 15.6% (n=12), 1.3% (n=1), and 18.2% (n=14) of the participants, respectively. The odds ratios (OR) for caloric and caloric + VEMP were as follows: (OR: 1.48 [95% CI: 0.48-5.48]) and (OR: 2.015 [95% CI: 0.56-7.25]), respectively. When the cut-point of blood pressure was set at > 140/90 (stage 2 HTN), having HTN-2 was associated with increased odds of abnormal caloric (OR:1.63 [95% CI: 0.46-5.77]) and caloric + VEMP measurements (OR: 1.56 [95% CI: 0.47-5.2]) (Table 2). Due to the limited overall sample size, the associations did not reach statistical significance; however, the results for each outcome within HTN status by HIV status were as expected (Table 2).

**Table 2:** Associations between HTN and caloric, VEMP, and Caloric + VEMP Outcomes were adjusted by age and HIV status by all HTN; and by only stage 2 HTN. Outcomes are odds ratios (OR) with 95% CI, and p-values. Hypertension (HTN), cervical vestibular evoked myogenic potentials (VEMP), human immunodeficiency virus (HIV), Odds Ratios (OR), confidence intervals (CI). P-value was set at 0.05 for statistical significance.

	Caloric	Caloric p-value	VEMP	VEMP p-value	Caloric + VEMP	Caloric + VEMP p-value
Associations between all HTN and VNG outcomes						
Age (per year increase)	OR: 1.01 (95% CI: 0.92-1.10)	p=8.17	OR: 1.00 (95% CI: 0.76-1.32)	p=0.997	OR: 1.04 (95% CI: 0.95-1.13)	p=0.43
HIV status (+ or -)	OR: 2.76 (95% CI: 0.32-23.59)	p=0.35	No estimate		OR: 3.32 (95% CI: 0.39-28.22)	p=0.27
HTN stages 1 and 2 combined vs No HTN	OR: 1.48 (95% CI: 0.48-5.48)	p=0.56	No estimate		OR: 2.01 (95% CI: 0.56-7.25)	p=0.28
Associations between Stage 2 HTN and VNG outcomes						
Age (per year increase)	OR: 1.00 (95% CI: 0.92 – 1.1)	p=0.96	OR: 1.03 (95% CI: 0.74 – 1.42)	p=0.87	OR: 1.03 (95% CI: 0.94 – 1.12	p=0.43
HIV status (+ or -)	OR: 2.8 (95% CI: 0.33 – 24.00)	p=0.35	No estimate		OR: 3.3 (95% CI: 0.39 – 27.9)	p=0.48
HTN stage 2 (vs No HTN + Stage 1 HTN)	OR: 1.63 (95%; CI: 0.46-5.77)	p=0.45	No estimate		OR: 1.56 (95% CI: 0.47-5.2)	p=0.51

## Discussion

This study found high ORs for vestibular impairment and HTN. This finding is consistent with previous evidence for a relationship between vestibular impairments and HTN [4]. As mentioned earlier, the lack of statistical significance is probably due to the relatively small sample size. Evidence in the literature shows that some disorders of the VNG are associated with HTN. For example, benign paroxysmal positional vertigo, central vertigo, Meniere’s disease, and vestibular vertigo have all been associated with HTN [8-12]. The body of literature on this topic is relatively small. This study adds to that literature and suggests the need for further research with larger samples using these common clinical tests. These data indicate that the assessment of dizziness is complex. Clinicians routinely test blood pressure but, except in primary care clinics, they may not necessarily relate the blood pressure level to the clinical problem. These data, and the previous evidence in the literature, support the need for clinicians to think in multidisciplinary terms when considering a patient’s complaint of dizziness.

The prevalence of HTN among PLWH is high [2]. HTN is a continuing problem in middle-aged people living with and without HIV. The evidence is unclear if HTN occurs more in PLWH than in PLWOH. One study showed that the frequency of HTN in the two groups did not differ [13] but another study, using a somewhat different paradigm, found a higher prevalence in PLWH [14]. The present study suggests that the relationship between HTN and vestibular impairments was greater among PLWH as compared to PLWOH for both HTN stages. 

To our knowledge, no previous study known has tested the relationship between HTN and vestibular impairments in PLWH. Heinze et al. did show a high prevalence of vestibular disorders in the HIV population [15], higher than our previous work has shown [15-17]. The two sets of studies differed in some technical aspects - none of those studies examined the relationship to HTN. The present study is, to our knowledge, the first test of this relationship. The English-language abstract of a study in Russian, which described only PLWOH who were hospitalized due to HTN, reported that 20% of patients with vertigo probably had neurologic or peripheral vestibular disorders without further suggesting the mechanism [18]. Other evidence suggests that HTN is related to central vestibular disorders [19].

The study had some limitations. The relatively small sample in the present study makes it akin to a pilot study. Investigating the etiology of this difference by HIV status would require a larger sample than was available to us. Such a sample would have to include relevant biomedical markers of HIV (i.e. anti-retroviral medication, immunological, virologic, and metabolic characterizations) [15]. Also, this study was performed retrospectively; a prospective study would have been more useful. Finally, the study cohort included only women. No such study has yet been done with men.

## Conclusions

We achieved the goal of the study in finding a relationship between vestibular impairment and HTN. The data from this study are the first evidence supporting the idea that some vestibular impairments may be related to HTN in HIV. This finding may explain why some complaints of patients are often difficult to understand. Because patients often cannot describe their sensory experiences clearly, patients and clinicians can easily conflate the sensations of vertigo and lightheadedness. This may be partly responsible for a large number of different clinician visits and tests that patients have to go through before being correctly diagnosed with vestibular impairments, resulting in high costs to the individual and the healthcare system. Clinicians should consider this issue when working up patients who complain of dizziness.
